# Converging Transmission Routes of the Highly Pathogenic Avian Influenza H5N1 Clade 2.3.4.4b Virus in Uruguay: Phylogeographic Insights into Its Spread Across South America

**DOI:** 10.3390/pathogens14080793

**Published:** 2025-08-08

**Authors:** Ana Marandino, Gonzalo Tomás, Yanina Panzera, Joaquín Williman, Filipe Zimmer Dezordi, Gabriel Luz Wallau, Sirley Rodríguez, Ramiro Pérez, Lucía Bassetti, Raúl Negro, Valeria Uriarte, Carmen Leizagoyen, Ruben Pérez

**Affiliations:** 1 Facultad de Ciencias, Universidad de la República, Montevideo 11400, Uruguay; amarandino@fcien.edu.uy (A.M.); gtomas@fcien.edu.uy (G.T.); ypanzera@fcien.edu.uy (Y.P.); jwilliman@fcien.edu.uy (J.W.); 2 Instituto Aggeu Magalhães (IAM)-Fundação Oswaldo Cruz (FIOCRUZ), Recife 50670-420, Brazil; zimmer.filipe@gmail.com (F.Z.D.); gabriel.wallau@fiocruz.br (G.L.W.); 3 Bernhard Nocht Institute for Tropical Medicine, National Reference Center for Tropical Infectious Diseases, WHO Collaborating Center for Arbovirus and Hemorrhagic Fever Reference and Research, D-20359 Hamburg, Germany; 4 División de Laboratorios Veterinarios “Miguel C. Rubino”, Dirección General de Servicios Ganaderos, Ministerio de Ganadería, Agriculturay Pesca, Montevideo 12100, Uruguay; srodriguez@mgap.gub.uy (S.R.); raperez@mgap.gub.uy (R.P.); lbassetti@mgap.gub.uy (L.B.); rnegro@mgap.gub.uy (R.N.); 5 Dirección Nacional de Biodiversidad y Servicios Ecosistémicos (DINABISE), Ministerio de Ambiente, Montevideo 11100, Uruguay; valeriau75@gmail.com (V.U.); carmenleizagoyen@gmail.com (C.L.)

**Keywords:** avian influenza, South America, genetic clades, mammal hosts, transmission routes, phylogeography

## Abstract

The highly pathogenic avian influenza H5N1 2.3.4.4b clade virus has caused widespread outbreaks across South America, primarily affecting seabirds, poultry, and marine mammals. The virus likely reached the continent through migratory birds from North America, initially spreading along the Pacific coast before advancing into Atlantic-bordering countries such as Argentina, Uruguay, and Brazil. This study investigated the dynamics of H5N1 strains in Uruguay during outbreaks from February and October 2023. We analyzed an updated South American database, including a newly sequenced viral genome from a royal tern (*Thalasseus maximus*) collected at the end of the outbreaks. Phylogeographic reconstruction revealed two distinct South American phylogroups comprising Uruguayan strains: one mainly driven by wild birds and poultry, with the royal tern strain clustering with Brazilian isolates, and another primarily associated with marine mammals, displaying adaptive residues in the PB2 protein. In Uruguay, these phylogroups delineate two main transmission routes: (i) an avian-derived pathway originating in Argentina and (ii) a pinniped-derived route from Chile. Brazil, initially colonized via the Argentine route, later emerged as a secondary source for Uruguay. This host-pathway interplay underscores the virus’s cross-species potential and highlights the need for coordinated regional surveillance within a One Health framework to mitigate zoonotic risks.

## 1. Introduction

The influenza A virus, a member of the family *Orthomyxoviridae* and the only species within the genus *Alphainfluenzavirus*, is a significant pathogen that affects both birds and mammals. A remarkable shift in the epidemiology of avian influenza occurred in 1996 with the emergence of the highly pathogenic H5 lineage [1]. Initially maintained mainly in domestic poultry, this lineage later became enzootic in wild birds and evolved into multiple genetically and antigenically distinct clades [2]. In 2020, clade 2.3.4.4b emerged and rapidly caused widespread outbreaks, devastating wild and domestic bird populations across Asia, Europe, Africa, and North America [3,4,5,6,7]. 

The ongoing panzootic caused by the highly pathogenic avian influenza (HPAI) H5N1 clade 2.3.4.4b virus is unprecedented not only because of its broad ecological impact but also for its successful transmission to mammals, including humans [8,9,10]. The reach of clade 2.3.4.4b has extended to polar regions, where it causes ecological disruption by affecting key species such as polar bears in the Arctic as well as marine mammals and penguins in Antarctic and sub-Antarctic islands [11,12,13]. 

The impact of the clade 2.3.4.4b intensified significantly in March 2024 when the B3.13 genotype—a reassortant of the European 2.3.4.4b and North American wild bird strains—emerged in dairy cows in Texas, USA [9,14]. Phylogenetic evidence indicates a single spillover event into cattle in late 2023 or early 2024, followed by transmission to other mammals and spillback into avian species [15]. This event resulted in human infections among farm workers during an outbreak at a Colorado poultry farm [16]. This scenario highlights the urgent need for coordinated responses based on a One Health approach, recognizing the link between animal, human, and environmental health [8]. Robust surveillance and genomic monitoring across ecosystems are essential to anticipate viral evolution, mitigate zoonotic risk, and guide public health interventions.

In South America, the HPAI H5N1 clade 2.3.4.4b virus emerged in 2023, leading to significant mortality among wild birds, including endangered species, and impacting the poultry industry in several Pacific-bordering countries [3,17,18,19]. The virus also caused unprecedented deaths in marine mammals and occasional infections in terrestrial carnivores [20,21,22,23]. Uruguay was the first to report the 2.3.4.4b clade in wild and backyard poultry along the Atlantic coast in 2023 [18]. The strain circulating among birds also affected South American coatis (*Nasua nasua*) in captivity, showing a 627K residue in the RNA polymerase basic protein 2 (PB2), an amino acid associated with early mammalian adaptation [21,24]. By August 2023, the virus was also detected in pinnipeds, which exhibited 591K and 701N residues in PB2 correlated with mammalian adaptations [22]. The strains transmitted by pinnipeds, which carry these mutations, subsequently spread to marine birds, introducing viruses with mammalian adaptive changes into avian hosts. 

Understanding the evolutionary dynamics and transmission routes of H5N1 in South America is essential to mitigate its ecological and public health impacts. This study examines an updated genome dataset of H5N1 strains from Uruguay, including a newly sequenced genome from the end of the outbreaks. By analyzing the Uruguayan strains within a South American context, we explore the virus’s genetic diversity and transmission patterns, clarify how transmission routes converge, and identify the factors that promote regional persistence and evolution.

## 2. Materials and Methods

### 2.1. Uruguayan Strains and New Sample Collection

Outbreaks in Uruguay occurred from February to October 2023. Strains from wild birds, backyard poultry, South American coatis, and marine mammals were previously collected by our group and are available in the GenBank database (Table S1). In this study, we included the most recent sequence obtained from the Uruguayan outbreaks in this dataset. This strain was isolated from a royal tern (*Thalasseus maximus*) found dead in October 2023 on Isla de Flores, an island located 17 km off the coasts of Montevideo and Canelones, Uruguay. The specimen was discovered during routine surveillance for avian influenza amid the 2023 outbreak in Uruguay, and clinical signs could not be determined. Only one royal tern was found, and no mass mortality events were reported. 

A brain tissue sample was stored in 3 mL of Dulbecco’s Modified Eagle’s Medium (Gibco™ DMEM. Thermo Fisher Scientific Inc., Pittsburgh, PA, USA), supplemented with 1% adult bovine serum and 2% antibiotic/antimycotic solution (Sigma-Aldrich, Inc., St. Louis, MO, USA). 

### 2.2. AIV Detection and Sequencing HA Subtyping

The detection and characterization of the influenza virus were performed as previously described [18,21,22]. Sequencing was conducted using an Illumina MiniSeq (Illumina, San Diego, CA, USA) with a MiniSeq™ Mid Output Reagent Cartridge (2 × 150 cycles). Raw data were trimmed and filtered using the BBDuk plugin in Geneious Prime 2020.2.1 software, and clean reads were mapped to a reference Uruguayan avian influenza genome (Accession: OR38159−2OR381599) with Minimap2 [25]. The assemblies were manually curated, and the consensus sequence was obtained and submitted to the GenBank database. 

### 2.3. Dataset

H5N1 avian influenza virus sequences were downloaded from GISAID [26] in December 2024. For our analysis, we retained only those sequences from South America that met the following criteria: (1) complete coding sequences for all segments, (2) low ambiguity content (i.e., few or no unresolved “N” nucleotides), and (3) classification as genotype B3.2.

Sequences were classified based on the similarity of the eight segments with previously categorized strains [21]. All segments were concatenated to improve phylogenetic resolution. The sequences were then trimmed to the start (ATG) and final stop codons and aligned using MAFFT v7 [27]. Metadata, including geographic coordinates and amino acids linked to mammalian adaptations (PB2: 591, 627, and 701), were recorded for all sequences.

### 2.4. Phylogenetic Reconstruction

Phylogeographic analysis was conducted using the Nextstrain CLI software (v8.5.4) [28]. Using the Augur toolkit (v27.0.0) [29], a maximum likelihood phylogeny was reconstructed with IQ-TREE 2 (v2.3.6) [30], starting with 1000 parsimonious trees (-ninit 1000) and branch support assessed using the approximate likelihood-ratio test (aLRT) with 1000 replicates (-alrt 1000). Automatic model selection was employed, and the GTR+F+R2 model was chosen. The resulting tree was then refined with TreeTime (v0.11.4) [31] through the “augur refine” command, applying an estimated constant coalescent rate that best fits the data (-coalescent opt) and inferring divergence dates for each internal node (-date-inference marginal). The tree was rooted using the sequence with the earliest sampling date (-root oldest).

## 3. Results

### 3.1. Detection and Sequencing of HPAI H5N1 in a Uruguayan Royal Tern 

On 25 October 2023, a dead royal tern was found during surveillance on Isla de Flores, Uruguay, and tested positive for the matrix gene and H5 subtype through RT-qPCR. The complete genome of this strain (M27-2023) was sequenced and deposited in GenBank (Accession: PV810114−PV810121). The HA and NA genes share over 99.8% nucleotide identity with South American HPAI H5N1 clade 2.3.4.4b strains from avian outbreaks, differing by only one or two nucleotides from Brazilian wild bird isolates.

### 3.2. Phylogeographic Analysis

**Figure 1:**
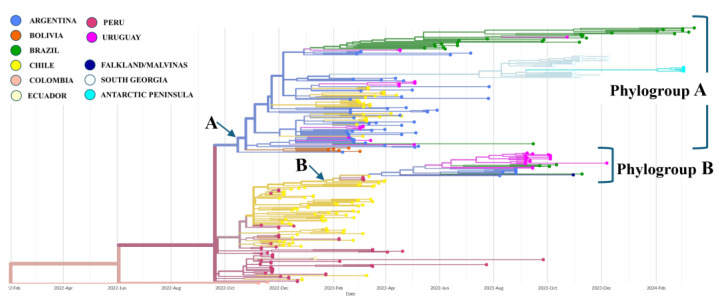
A phylogenetic tree showing all examined South American strains (H5N1 2.3.4.4b clade). Tip colors indicate their country of origin. Uruguayan samples are found in the A and B phylogroups with strong aLRT branch support (A: 92.6, B: 97.6). In addition to South American samples, phylogroup A also includes sequences from South Georgia Island and the Antarctic Peninsula. Phylogroup B includes a sequence from the Falkland/Malvinas Islands.

The phylogenetic tree of concatenated coding sequences of South American B3.2 genotype strains revealed two phylogroups (A and B) with robust branch support (A: 92.6, B: 97.6) (Figure 1 and Figure S1).

**Figure 2:**
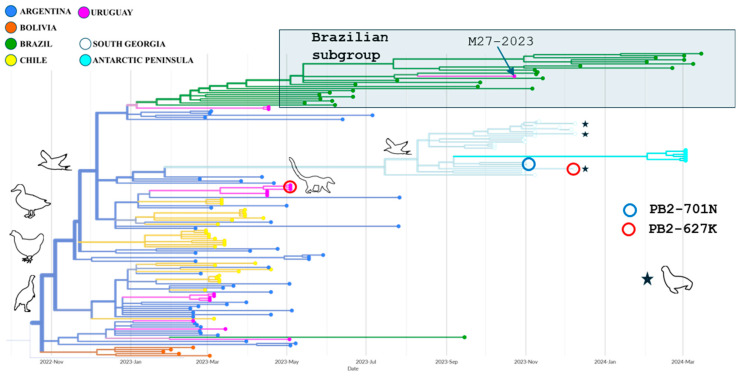
Close-up of phylogroup A from the phylogenetic analysis. Phylogroup A includes the M27-2023 strain from a royal tern sampled in Uruguay. This group has strong aLRT branch support (92.6) and mainly consists of strains from wild birds and poultry, although three strains are from coatis and three are from pinnipeds (stars) on South Georgia Island. The PB2 protein of two coati-derived strains and one pinniped contains the 627K substitution (red circles), whereas a wild bird isolate from South Georgia Island exhibits the 701N residue (blue circle).

Phylogroup A, which includes sixteen Uruguayan samples (Table 1), such as the recently obtained M27-2023 from a royal tern, clusters with strains from Argentina, Bolivia, Brazil, Chile, South Georgia Island, and the Antarctic Peninsula (Figure 2 and Figure S1). It most likely originated in Argentina, with a 99% probability, in October 2022, with poultry serving as the ancestral hosts (100% probability). Most South American strains are from birds, except for three strains from Uruguayan coatis. The other non-avian hosts were pinnipeds from South Georgia Island (Antarctic region). The M27-2023 strain belongs to a Brazilian subgroup (originating in January 2023) of wild birds, with two basal sequences from backyard poultry in Uruguay (Figure 2). This clade maintains typical avian amino acid profiles, apart from the 627K mutation found in two coatis and a South Georgia Island pinniped and the 701N residue observed in a South Georgia Island wild bird (Figure 2). The 627K and the 701N amino acids in coatis, pinnipeds, and the wild bird occurred in different branches of phylogroup A.

**Figure 3:**
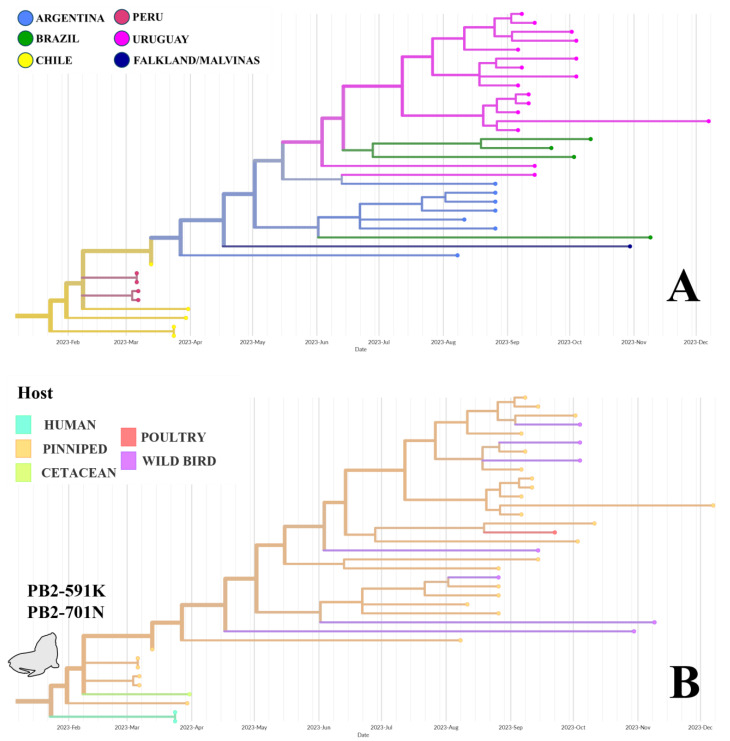
Close-up of phylogroup B (aLRT branch support = 97.6) from the phylogenetic analysis by country (**A**) and host (**B**). South American tern hosts are dispersed among strains from Argentina and Uruguay (**A**). This group, which has ancestral pinniped hosts (**B**), is characterized by PB2 residues 591K and 701N, and also includes wild birds and human samples.

Phylogroup B, which includes sixteen Uruguayan samples (Table 1), originated in Chile, with a 100% probability, in January 2023. This group also contains strains from Peru, Chile, Argentina, and Brazil (Figure 3A and Figure S1). A strain from a wild bird within this group was collected in the Falkland/Malvinas Islands (sub-Antarctic region). Pinnipeds are the primary and ancestral hosts, with a 98% probability, while wild birds, poultry, and humans are derived and less frequently observed (Figure 3B). Strains from wild birds, especially South American terns (*Sterna hirundinacea*), are intermixed with pinniped strains. All group B strains from pinnipeds, wild birds, and humans exhibit the mammalian adaptation mutations Q591K and D701N in PB2, which always occur together (Figure 3B). Three basal strains of this group, two from pinnipeds and one from a wild bird, have only the D701N substitution. 

### 3.3. Visualization of Virus Spread

The Nextstrain analysis provides a detailed timeline of the spread of the HPAI H5N1 2.3.4.4b clade virus across South America, revealing multiple independent introductions into Uruguay (Video S1). The spread of the virus followed a trajectory from Colombia to Peru, then to Argentina and Chile. 

Phylogroup A expanded from Argentina to Bolivia and Uruguay, later reaching Brazil and eventually re-entering Uruguay. This phylogroup also dispersed southward from Argentina to Antarctic regions, including South Georgia Island and the Antarctic Peninsula. These transmission routes were primarily mediated by migratory birds, although pinnipeds were occasionally affected during the later stages of the epidemic.

Phylogroup B emerged in marine mammals along the Chilean coast and then spread through Argentina and Uruguay before reaching the coastal regions of Brazil. This transmission pathway was predominantly associated with pinnipeds but also involved seabirds and, more rarely, humans and domestic poultry.

## 4. Discussion

The period between late 2022 and 2023 marked the first-ever invasion of South America by HPAI H5N1 clade 2.3.4.4b, with confirmed detections in at least 83 wild bird species and 11 wild mammal species across Chile, Peru, Colombia, Ecuador, Venezuela, Uruguay, Argentina, Brazil, Bolivia, and Paraguay. Several species experienced mass mortality exceeding 10% of their populations, including 11.8% in the neotropical cormorant (*Nannopterum brasilianus*), 17.1% in the Humboldt penguin (*Spheniscus humboldti*), 20.1% in the Peruvian booby (*Sula variegata*), 14.9% in the South American sea lion (*Otaria flavescens*), and as high as 61.9% in the Peruvian pelican (*Pelecanus thagus*) [19,32]. In the most severe case, Southern elephant seal pups (*Mirounga leonina*) experienced an estimated 97% mortality rate (over 17,000 deaths), representing the largest recorded mortality event for this species [33]. Beyond these substantial population losses, the epizootic has likely caused widespread, yet unquantified, disruptions to social structures, reproductive dynamics, and interspecific interactions, raising serious concerns about long-term ecological consequences [34].

The South American invasion was mediated by strains belonging to the B3.2 genotype, a reassortant of North American origin that combines the PA, HA, NA, and M segments of Eurasian wild bird ancestry with the PB2, PB1, NP, and NS segments from North American bird lineages [5]. Other genotypes have also been introduced sporadically, such as B2.2 (five Venezuelan strains) and B1.3 (three Colombian strains), although B3.2 remains dominant across the region [21,35]. 

Unlike North America, where reassortment is frequent with co-circulating avian influenza (LPAI) strains [10], only the emergence of one reassortment event has been documented in South America [36]. This H5N1 reassortment took place during an Argentine outbreak in 2025 and involved acquiring four segments (PB2, PB1, PA, and NS) from a locally circulating LPAI virus. The comparatively lower frequency of reassortment events that emerged in South America may reflect a single primary introduction of H5N1 in the region, unlike the multiple incursions from Europe and Asia observed in North America [5]. Additionally, limited genomic surveillance in South America relative to North America [37] may contribute to leaving reassortment events undetected.

**Figure 4:**
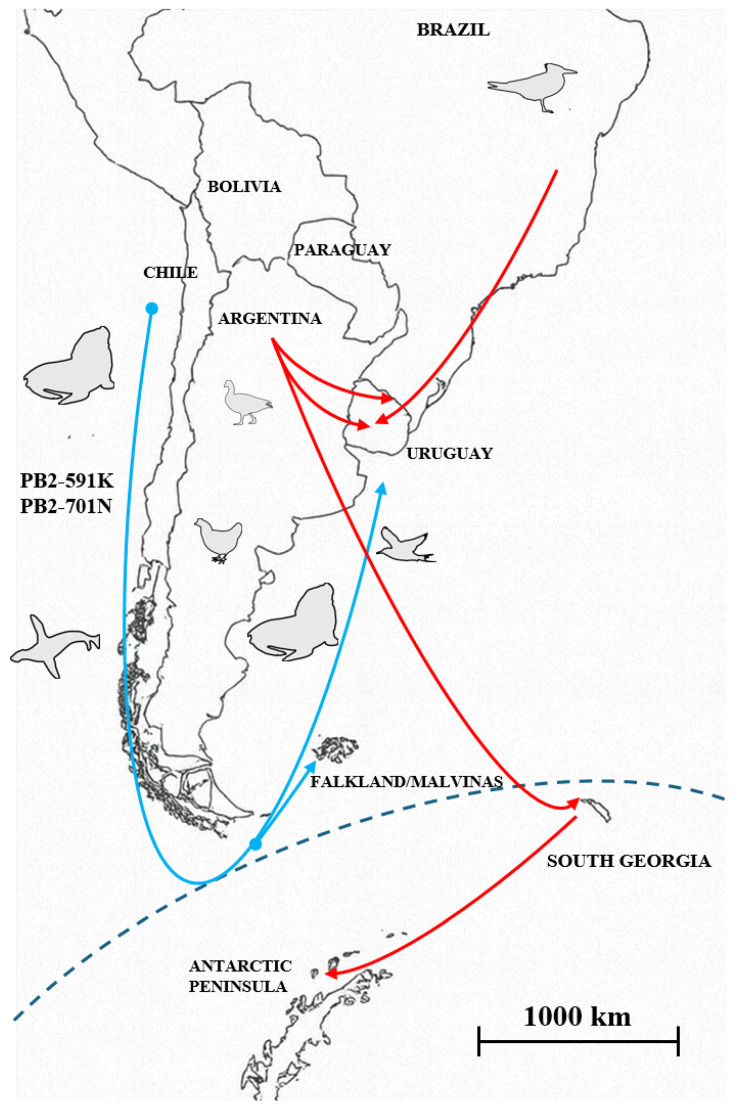
The convergence of migration routes in Uruguay. Uruguayan strains (phylogroup A, red lines) mainly originated from birds, arriving in February and May 2023 from Argentina. Additionally, a secondary entry was recorded from Brazil in August 2023. Phylogroup A strains also reached South Georgia Island and subsequently the Antarctic Peninsula. Phylogroup B strains (blue line), primarily originating from and mainly transmitted by pinnipeds, reached Uruguay in August 2023; these strains also entered the Falkland/Malvinas Islands (sub-Antarctic region).

The predominant B3.2 genotype probably entered South America via Colombia. From there, it spread to Peru, Chile, and Argentina, establishing central hubs that helped its dispersal across the continents and to Antarctic and sub-Antarctic islands. In Uruguay, phylogenetic clustering indicates multiple introductions of B3.2, resulting in two distinct clades defined by their geographic origin, host species, and adaptive mutations in the PB2 gene (Figure 1 and Figure 4, Video S1). 

### 4.1. Avian-Derived Transmission (Phylogroup A)

Phylogroup A emerged in Argentina, which became a significant hub for transmission, enabling the further spread to Bolivia, Chile, Uruguay, and Brazil. Although primarily affecting poultry and wild birds, sporadic cases in mammals are observed (Figure 3B and Figure S1). Infected birds of phylogroup A showed neurological signs (ataxia, twitching), behavioral changes (lethargy, anorexia), and hemorrhagic lesions, often resulting in acute mortality [11,18].

The South American phylogroup A was also linked to outbreaks in South Georgia Island and the Antarctic Peninsula (Figure 4, Video S1). South Georgia, located about 1400 km east-southeast of the South American mainland and south of the Antarctic Polar Front, is considered part of the Antarctic region and provides critical habitat for penguins, seals, and seabirds. The Antarctic Peninsula, located about 1000 to 1300 km south of the continent, is the closest Antarctic landmass to South America (Figure 4).

The basal strain of phylogroup A originated from a wild bird (*Chloephaga melanoptera*) collected in the Jujuy Province (A/Goose/Argentina/389-1/2023), in the high-altitude Andean region known as the Puna in northwestern Argentina, close to the Bolivian border [38]. This was the first HPAI H5N1 clade 2.3.4.4b case detected in Argentina and suggests that wild birds may have contributed to the spread of the disease to poultry and become a key source for Bolivia, Uruguay, and Brazil. 

The M27-2023 strain from the royal tern originated from a secondary hub in Brazil, indicating independent introduction for phylogroup A from Argentina and Brazil.

Some members of phylogroup A occasionally harbor mammalian adaptive changes in PB2 (Figure 2). Samples from two Uruguayan coatis displayed the E627K substitution, which has been observed in Spanish and Finnish mink farms as well as in other carnivores [21,24,39]. This mutation has also been noted in viral RNA extracted from mammary gland tissue and milk samples from cows following experimental infection with the B3.13 genotype [40]. Furthermore, it has been found in a human case involving direct contact with infected dairy cattle [41]. The E627K mutation in the PB2 sequence may facilitate replication in mammals, including humans, and emphasizes the potential for adaptation. Notably, a pinniped from South Georgia Island has the 627K mutation, which emerged independently from that found in the Uruguayan coatis. A wild bird from the same island displayed the 701N mutation, another well-known adaptive change. The 627K and 701N generally emerge independently, supporting parallel adaptive pathways in mammalian hosts [42].

### 4.2. Pinniped-Derived Transmission (Phylogroup B)

Phylogroup B originated in Chile and spread along the Pacific coast to Patagonia and the Atlantic coast, reaching Argentina, Uruguay, and Brazil (Figure 3 and Figure 4). Most strains have been found in sea lions (*Otaria flavescens*) and other marine mammals, highlighting their role as key hosts in the transmission dynamics of this group. Unlike phylogroup A, which mainly includes strains from birds, phylogroup B emerged in marine mammals and shows adaptive mutations, indicating ongoing adaptation to non-avian hosts. All phylogroup B strains share two mammalian-adaptive substitutions in PB2 (Q591K, D701N). This unusual combination of mammalian adaptations in PB2 has been previously observed in South American pinnipeds [19,22,32,43] and likely represents a set of co-adapted changes. Two ancestral strains from pinnipeds, which are phylogenetically basal but not part of phylogroup B, only carry the 701N residue, suggesting that this substitution appeared before the emergence of 591K. These findings support a stepwise evolutionary pathway leading to the co-occurrence of both amino acid substitutions.

During the spread of phylogroup B, wild birds cohabiting with coastal pinnipeds in Peru, Chile, and Argentina acquired the Q591K and D701N PB2 substitutions, with no evidence of reversion events (Figure 4). These residues have also been found in poultry over 1000 km inland, suggesting that bird-mediated dispersal of mammal-adapted strains can reach commercial farms [44]. In this context, mapping the genetic variability of avian influenza viruses is crucial, as intensive poultry farming promotes viral amplification, persistence, and reassortment among co-circulating strains [41]. High-density flocks and frequent movements of birds, whether for trade, live bird markets, or exhibitions, facilitate viral replication and transmission, creating optimal conditions for co-infections and the emergence of novel genotypes [5,36,43]. In South America, poultry operations are concentrated in specific areas, often adjacent to wetlands that host dense wild bird populations, further increasing the risk of reassortment events. Under these ecological and production conditions, commercial poultry farms function as hotspots for viral diversification, thereby increasing the zoonotic and pandemic risks linked to HPAI viruses.

The range of phylogroup B extended to the Falkland/Malvinas Islands through the southern fulmar (*Fulmarus glacialoides*), which contains the Q591K and D701N mutations, underscoring its potential for long-distance dispersal and amplification risks [11]. The Falkland/Malvinas Islands are situated in the South Atlantic Ocean, approximately 500 km east of the Patagonian coast of Argentina, a highly productive marine ecosystem that supports numerous migratory seabirds and marine mammals. These islands lie north of the Antarctic Polar Front and are sometimes regarded as a sub-Antarctic archipelago [11].

### 4.3. Mammalian Adaptation and One Health Risk

Substitutions such as E627K, Q591K, and D701N in PB2 enhance mammal-to-mammal transmission, potentially increasing the virus’s risk [19,21,45,46,47,48,49]. However, the viruses currently circulating in South America lack specific amino acid residues in the HA sequence that increase the affinity for human receptors, as well as other key mutations associated with higher transmission [50]. The distribution of PB2 adaptive mutations (627K versus 591K+701N) differs between phylogroups A and B, supporting previous findings that suggest distinct evolutionary origins and adaptive pathways enhancing replication in mammalian hosts. Despite these adaptive signatures, the overall risk to human health remains low [51]. Nevertheless, the virus’s ability for rapid adaptive genetic change, through both antigenic drift and shift, raises concerns about its potential pandemic risk. These findings underscore the need for continuous genomic surveillance and the implementation of integrated One Health approaches.

### 4.4. Uruguayan Dynamics: Role of Pinnipeds and Birds

Our findings suggest that Uruguay served as a convergence point for two main transmission routes: (i) the avian-derived pathway, linked to phylogroup A, originated in Argentina, and (ii) the pinniped-derived route, related to phylogroup B, from Chile to the Atlantic coast (Figure 4). The royal tern M27-2023 strain, which belongs to the avian-derived pathway of phylogroup A, originates from a secondary Brazilian hub of the avian-derived pathway.

Uruguayan South American terns (*Sterna hirundinacea*) and royal terns (*Thalasseus maximus*) participate in the transmission routes of phylogroups A and B, respectively, reflecting the intersection between host ecology and viral dispersal. Both species were sampled in Uruguay between August and September 2023 (Table 1; Figure 4). Although these species share broad distributions along the Atlantic coast of South America, their migratory behaviors differ [52,53]. Royal terns migrate northward during the austral winter from Uruguay and Argentina to Brazil, which explains how they can be infected by a Brazilian wild bird strain before returning to Uruguay. In contrast, South American terns breed along the Pacific coast of Chile and southern Argentina, migrating extensively to Uruguay and Brazil from November to April. The overlap of South American terns with pinniped populations along both coasts suggests they may have acquired infection via the pinniped-derived phylogroup B route, highlighting their potential role in interconnecting marine and avian transmission cycles.

## 5. Conclusions

Avian influenza dynamics in Uruguay result from the convergence of hosts, transmission routes, and viral genetic variability across terrestrial and aquatic environments, demonstrating the virus’s ability to spread intercontinentally through avian and marine pathways. Understanding these dynamics is crucial for improving surveillance and response efforts. Given the cross-border nature of H5N1 and its potential zoonotic impact, regional cooperation and the integration of veterinary and public health data are vital. Effective strategies should focus on monitoring migratory birds and marine mammals, implementing robust biosecurity measures in poultry farms, and establishing effective human surveillance systems to prevent the spread of influenza. This study underscores the value of a One Health framework to monitor cross-species transmission and anticipate zoonotic threats at the human–animal-environment interface.

## Figures and Tables

**Table 1:** HPAI H5N1 clade 2.3.4.4b virus strains from Uruguay. Collection dates, host species, phylogenetic groups (A and B) identified in this study, and adaptive residues in the PB2 protein are shown.

Strain	Date	Host Species	Phylogroup	PB2-591	PB2-701	PB2-627
014-M3	Feb 2023	*Cygnus melancoryphus*	A	Q	D	E
040-M5	Mar 2023	*Gallus gallus*	A	Q	D	E
040-M7	Mar 2023	*Gallus gallus*	A	Q	D	E
047-M1	Mar 2023	*Gallus gallus*	A	Q	D	E
047-M3	Mar 2023	*Gallus gallus*	A	Q	D	E
078-M2	Mar 2023	*Cygnus melancoryphus*	A	Q	D	E
124-M1	Apr 2023	*Gallus gallus*	A	Q	D	E
124-M3	Apr 2023	*Meleagrus gallopavo*	A	Q	D	E
124-M6	Apr 2023	*Meleagrus gallopavo*	A	Q	D	E
127-M4	Apr 2023	*Gallus gallus*	A	Q	D	E
127-M1	Apr 2023	*Gallus gallus*	A	Q	D	E
144-M3	May 2023	*Gallus gallus*	A	Q	D	E
146-M1	May 2023	*Nasua nasua*	A	Q	D	E
145-M2	May 2023	*Nasua nasua*	A	Q	D	K
145-M1	May 2023	*Nasua nasua*	A	Q	D	K
M27-2023	Oct 2023	*Thalasseus maximus*	A	Q	D	E
P6_6923	Sep 2023	*Otaria flavescens*	B	K	N	E
P7_6923	Sep 2023	*Otaria flavescens*	B	K	N	E
P5_6923	Sep 2023	*Otaria flavescens*	B	K	N	E
P4_6923	Sep 2023	*Otaria flavescens*	B	K	N	E
P10_8923	Sep 2023	*Otaria flavescens*	B	K	N	E
P8_8923	Sep 2023	*Arctocephalus australis*	B	K	N	E
P13_11923	Sep 2023	*Otaria flavescens*	B	K	N	E
P14_11923	Sep 2023	*Otaria flavescens*	B	K	N	E
P16_14923	Sep 2023	*Sterna hirundinacea*	B	K	N	E
P17_14923	Sep 2023	*Otaria flavescens*	B	K	N	E
P18_14923	Sep 2023	*Otaria flavescens*	B	K	N	E
P15_14923	Sep 2023	*Otaria flavescens*	B	K	N	E
P26_21023	Oct 2023	*Arctocephalus australis*	B	K	N	E
P23_41023	Oct 2023	*Sterna hirundinacea*	B	K	N	E
P24_41023	Oct 2023	*Sterna hirundinacea*	B	K	N	E
P25_41023	Oct 2023	*Sterna hirundinacea*	B	K	N	E

## Data Availability

The sequences that were obtained are available in GenBank (Accession: PV810114−PV810121).

## Supplementary Materials

The following supporting information can be downloaded at https://www.mdpi.com/article/10.3390/pathogens14080793/s1: Figure S1: A phylogenetic tree displaying the labels of all analyzed South American, Antarctic Peninsula, and sub-Antarctic strains. Video S1: Animation showing the spread of highly pathogenic avian influenza (HPAI) H5N1 across South America, with multiple introduction events highlighted in Uruguay. The animation was created using the interactive visualization tools provided by the Nextstrain platform (https://nextstrain.org/, accessed on 1 June 2025). Table S1: HPAI H5N1 clade 2.3.4.4b virus strains from Uruguay. Collection dates, hosts, phylogenetic groups (A and B) identified in this study, adaptive residues in the PB2 protein, and GenBank accession numbers are shown.
