# Supplementary material for: Converging Transmission Routes of the Highly Pathogenic Avian Influenza H5N1 Clade 2.3.4.4b Virus in Uruguay: Phylogeographic Insights into Its Spread Across South America
Source: Pathogens. 2025 Aug 8;14(8):793. doi: 10.3390/pathogens14080793 (PMC12389478; doi:10.3390/pathogens14080793)
Supplement: Supplementary file 1 [file pathogens-14-00793-s001.zip › Supplementary Figure S1.pdf]

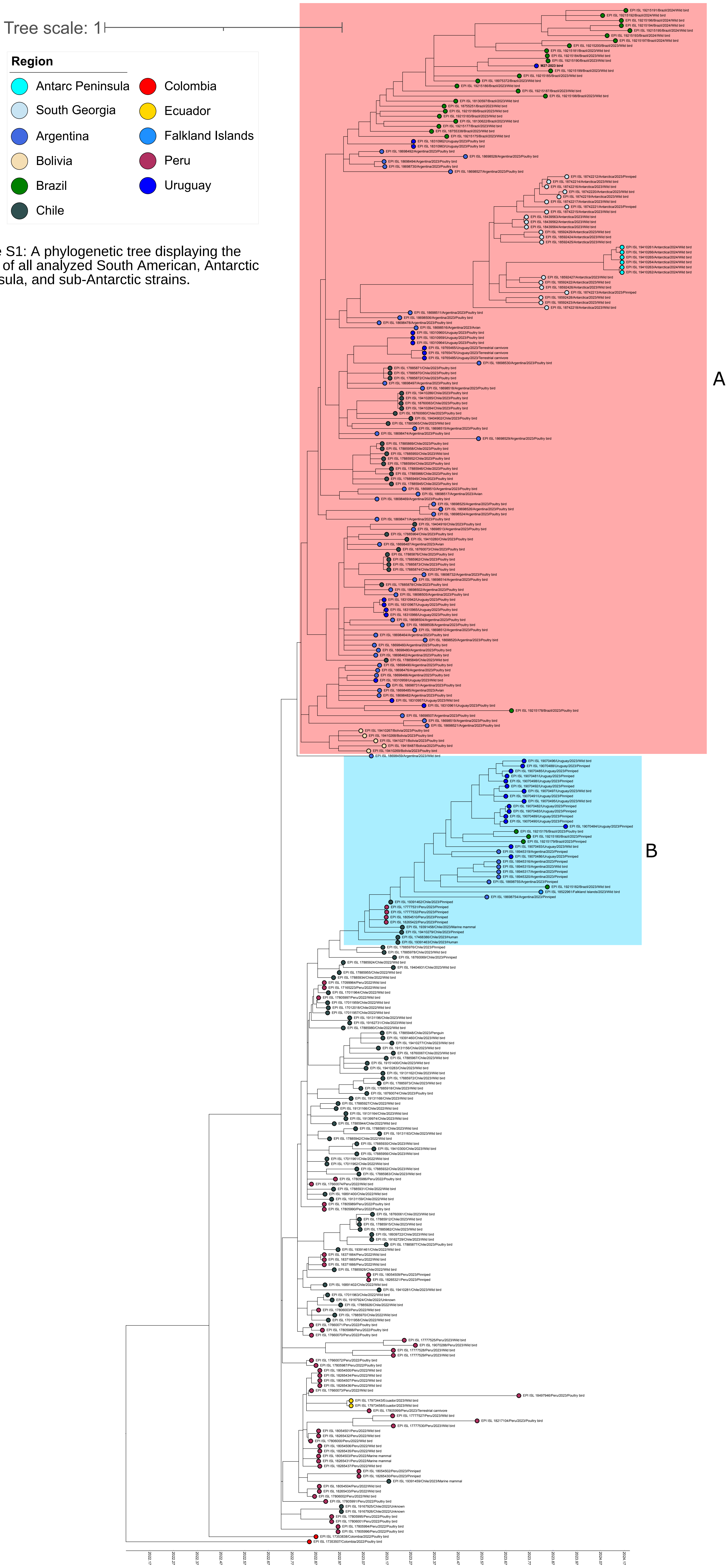

Figure S1: A phylogenetic tree displaying the labels of all analyzed South American, Antarctic Peninsula, and sub-Antarctic strains.
