# Supplementary material for: Converging Transmission Routes of the Highly Pathogenic Avian Influenza H5N1 Clade 2.3.4.4b Virus in Uruguay: Phylogeographic Insights into Its Spread Across South America
Source: Pathogens. 2025 Aug 8;14(8):793. doi: 10.3390/pathogens14080793 (PMC12389478; doi:10.3390/pathogens14080793)
Supplement: Supplementary file 1 [file pathogens-14-00793-s001.zip › Table S1.pdf]

Supplementary Table S1. HPAI H5N1 clade 2.3.4.4b virus strains from Uruguay. Collection dates, hosts, phylogenetic groups (A and B) identified in this study, adaptive residues in the PB2 protein, and GenBank accession numbers are shown.

Strain	Date	Host species	Phylogroup	PB2-591	PB2-701	PB2-627	Accession number
014-M3	Feb 2023	*Cygnus melancoryphus*	A	Q	D	E	OR381584–OR381591
040-M5	Mar 2023	*Gallus gallus*	A	Q	D	E	OR381592–OR381599
040-M7	Mar 2023	*Gallus gallus*	A	Q	D	E	OR381600–OR381607
047-M1	Mar 2023	*Gallus gallus*	A	Q	D	E	OR381608–OR381615
047-M3	Mar 2023	*Gallus gallus*	A	Q	D	E	OR381616–OR381623
078-M2	Mar 2023	*Cygnus melancoryphus*	A	Q	D	E	OR381672–OR381679
124-M1	Apr 2023	*Gallus gallus*	A	Q	D	E	OR381624–OR381631
124-M3	Apr 2023	*Meleagrus gallopavo*	A	Q	D	E	OR381656–OR381663
124-M6	Apr 2023	*Meleagrus gallopavo*	A	Q	D	E	OR381664–OR381671
127-M4	Apr 2023	*Gallus gallus*	A	Q	D	E	OR381640–OR381647
127-M1	Apr 2023	*Gallus gallus*	A	Q	D	E	OR381632–OR381639
144-M3	May 2023	*Gallus gallus*	A	Q	D	E	OR381648–OR381655
146-M1	May 2023	*Nasua nasua*	A	Q	D	E	PP426580−PP426587
145-M2	May 2023	*Nasua nasua*	A	Q	D	K	PP426572−PP426579
145-M1	May 2023	*Nasua nasua*	A	Q	D	K	PP426564− PP426571
M27-2023	Oct 2023	*Thalasseus maximus*	A	Q	D	E	PV810114−PV810121
P6_6923	Sep 2023	*Otaria flavescens*	B	K	N	E	OR912248, OR912270, OR912283, OR912299, OR912311, OR912324, OR912338
P7_6923	Sep 2023	*Otaria flavescens*	B	K	N	E	OR912249, OR912271, OR912284, OR912300, OR912312, OR912325, OR912339
P5_6923	Sep 2023	*Otaria flavescens*	B	K	N	E	OR912247, OR912269, OR912282, OR912298, OR912310, OR912323, OR912337
P4_6923	Sep 2023	*Otaria flavescens*	B	K	N	E	OR912351, OR912352, OR912353, OR912354, OR912355, OR912356, OR912357, OR912358

P10_8923	Sep 2023	*Otaria flavescens*	B	K	N	E	OR912251, OR912262, OR912273, OR912286, OR912302, OR912314, OR912327, OR912341
P8_8923	Sep 2023	*Arctocephalus australis*	B	K	N	E	OR912250, OR912261, OR912272, OR912285, OR912301, OR912313, OR912326, OR912340
P13_11923	Sep 2023	*Otaria flavescens*	B	K	N	E	OR912252, OR912263, OR912274, OR912287, OR912303, OR912315, OR912328, OR912342
P14_11923	Sep 2023	*Otaria flavescens*	B	K	N	E	OR912253, OR912264, OR912275, OR912288, OR912304, OR912316, OR912329, OR912343
P16_14923	Sep 2023	*Sterna hirundinacea*	B	K	N	E	OR912255, OR912265, OR912276, OR912296, OR912318, OR912331, OR912345
P17_14923	Sep 2023	*Otaria flavescens*	B	K	N	E	OR912256, OR912266, OR912277, OR912291, OR912306, OR912319, OR912332, OR912346
P18_14923	Sep 2023	*Otaria flavescens*	B	K	N	E	OR912257, OR912267, OR912278, OR912292, OR912307, OR912320, OR912333, OR912347
P26_21023	Oct 2023	*Arctocephalus australis*	B	K	N	E	OR912260, OR912281, OR912295, OR912309, OR912322, OR912336, OR912350
P23_41023	Oct 2023	*Sterna hirundinacea*	B	K	N	E	OR912359, OR912360, OR912361, OR912362, OR912363, OR912364, OR912365, OR912366
P24_41023	Oct 2023	*Sterna hirundinacea*	B	K	N	E	OR912258, OR912279, OR912293, OR912297, OR912334, OR912348
P25_41023	Oct 2023	*Sterna hirundinacea*	B	K	N	E	OR912259, OR912268, OR912280, OR912294, OR912308, OR912321, OR912335, OR912349
P15_14923	Sep 2023	*Otaria flavescens*	B	K	N	E	OR912254, OR912289, OR912305, OR912317, OR912330, OR912344
